# TAGLN-RhoA/ROCK2-SLC2A3-mediated Mechano-metabolic Axis Promotes Skin Fibrosis

**DOI:** 10.7150/ijbs.104484

**Published:** 2025-01-01

**Authors:** Xinwei Cheng, Zhen Gao, Jin Zhang, Hongkun Zheng, Shengzhou Shan, Jia Zhou

**Affiliations:** Department of Plastic and Reconstructive Surgery, Shanghai Ninth People's Hospital, Shanghai Jiao Tong University School of Medicine, 639 Zhizaoju Road, Shanghai, China.

**Keywords:** skin fibrosis, fibroblast, TAGLN, mechano-metabolic axis, glycolysis

## Abstract

Skin fibrotic diseases are characterized by abnormal fibroblast function and excessive deposition of extracellular matrix. Our previous single-cell sequencing results identified an enriched fibroblast subcluster in skin fibrotic tissues that highly expresses the actin cross-linking cytoskeletal protein Transgelin (TAGLN), which bridges the mechanical environment of tissues and cellular metabolism. Therefore, we aimed to investigate the role of TAGLN in the pathogenesis of skin fibrosis. Transwell, wound healing, collagen gel contraction assay, immunofluorescence and RNA-seq analyses were used to validate and explore the potential mechanisms of the TAGLN-RhoA/ROCK2-SLC2A3-mediated mechano-metabolic axis in dermal fibrosis. The therapeutic efficacy of targeting TAGLN was validated using a bleomycin-induced mouse model of skin fibrosis. Functional assays revealed that downregulation of TAGLN inhibited motility and secretory function of fibroblasts, including invasion, migration, contraction, and collagen secretion. The glucose carrier SLC2A3 was identified as one of the downstream targets of TAGLN by RNA-sequencing analysis and further validation. We further demonstrated that TAGLN regulates the expression of SLC2A3 through the RhoA/ROCK2 pathway, a key pathway of mechanotransduction, thereby affecting glycolysis and motility of fibroblasts. This study reveals the existence of the TAGLN-RhoA/ROCK2-SLC2A3 mechano-metabolic axis in skin fibrotic diseases and provides a promising target for its clinical treatment.

## Introduction

Skin fibrosis, including hypertrophic scars and keloids, is a fibroproliferative disorder characterized by abnormal fibroblast function and excessive deposition of extracellular matrix (ECM) 1,2. Skin fibrotic diseases not only lead to impaired appearance but also induce dysfunction, thereby severely affecting patient's social function and life quality. Current treatments for skin fibrotic diseases, including topical medications, steroid injections, cryotherapy, radiotherapy, and surgery, have limited efficacy and can hardly achieve satisfaction 3. Therefore, it is important to further investigate the molecular mechanisms of skin fibrosis and develop a promising therapeutic approach. In solid tumors, the cytoskeleton serves as a bridge between the mechanical environment of the tissue and cellular metabolism 4,5. Cytoskeletal protein allows tumor-associated fibroblasts to adapt to the variable mechanical microenvironment and to regulate energy production and movement accordingly 6. So far, it has been shown that altered mechanical forces and metabolic imbalance are the key components involved in the progression of skin fibrosis 7,8. However, in skin fibrotic diseases, it remains unknown whether there is a link between mechanical forces and metabolism to promote the progression of fibrosis.

In our previous single-cell sequencing results (GSE243716), we found a fibroblast subcluster enriched in human pathological scars (fibrotic skin tissues) with high expression of Transgelin (TAGLN). TAGLN is an actin cross-linking protein that senses changes in mechanical forces, stabilizes the cytoskeleton through actin binding, and plays a role in cell differentiation, cell migration, cell invasion, and matrix remodeling 9-11. Therefore, we speculated that TAGLN plays a key role in skin fibrosis and further investigated its mechanism. Here, we reported the role of a mechano-metabolic axis consisting of TAGLN-RhoA/ROCK2-SLC2A3 in driving skin fibrosis.

We found that TAGLN was upregulated in human fibrotic skin tissues and identified the glucose carrier protein SLC2A3 as a downstream molecule of TAGLN by bulk RNA sequencing. We further verified that TAGLN upregulated SLC2A3 expression through the activation of the RhoA/ROCK2 pathway, which in turn enhanced fibroblast motility and glycolysis. Overall, overexpression of TAGLN affects metabolic homeostasis and enhances glycolysis through the activation of SLC2A3 via the RhoA/ROCK2 pathway, which promotes the progression of skin fibrosis. In conclusion, targeting TAGLN may represent a viable strategy for breaking the link between mechanotransduction and metabolic homeostasis in skin fibrotic diseases.

## Materials and Methods

### Patient samples

In accordance with the principles of the Declaration of Helsinki and with the ethical approval of the Local Human Research Ethics Committee of Shanghai Jiao Tong University School of Medicine, 12 normal skin tissue samples, 15 hypertrophic scars samples, and 15 keloid tissue samples were collected from January to October 2023 during plastic surgery procedures at the Shanghai Ninth People's Hospital (Supplementary Table S1). These specimens were used to perform extraction of primary cells and tissue sections. Extracted primary cells were used for all subsequent cell experiments.

### Primary cell isolation and culture

Primary human dermal fibroblasts were isolated from skin samples (including normal skin, hypertrophic scars, and keloids) provided by Shanghai Ninth People's Hospital. After trimming off the subcutaneous adipose tissue, 0.3cm*0.3cm squares were scratched on the epidermis of the tissue. The skin tissue was then placed in 3mg/ml of ROCHE Dispase II (Sigma-Aldrich, USA) at 4°C overnight to dissociate the epidermis. The following day, the epidermis was removed with forceps, and then tissue was further minced with scissors until they reached a pasty consistency. Subsequently, the tissue was transferred to a solution containing 3mg/ml collagenase NB4 (Nordmark, Germany), 4-5 times the volume of the tissue, and incubated at 37°C for 8-10 hours. The liquid was then passed through a 200-mesh sieve, and the filtrate was centrifuged at 1200 rpm/min for 10 minutes. After discarding the supernatant, the precipitate was resuspended in sterile PBS and centrifuged again at 1200 rpm/min for 5 minutes. Following the removal of the supernatant, the cells were resuspended in Dulbecco's Modified Eagle Medium (Gibco, USA) Dulbecco's Modified Eagle Medium (DMEM) (Gibco, USA) supplemented with 10% fetal bovine serum and antibiotics (100 IU/mL penicillin and 100 mg/mL streptomycin). The cells were cultured in dishes and maintained in a cell culture incubator at 37°C with 5% CO2. Passage was performed whenever cell density reached 75%-90%, using trypsin for digestion, with a passage ratio of 1:3 12,13. Primary cells from passages 3-6 were utilized for cytological experiments.

Passage was performed whenever cell density reached 75%-90%, using trypsin for digestion, with a passage ratio of 1:3. Primary cells from passages 3-6 were utilized for cytological experiments. The cells were subjected to immunofluorescence staining for Vimentin to verify their fibroblast identity.

### Purification and quantitative real-time PCR (RT-qPCR)

The total RNA of the samples was extracted using an EZ-press RNA Purification Kit (EZBioscience, USA). According to the manufacturer's instructions, reverse transcription was performed using Reverse Transcription Master Mix (EZBioscience, USA). The samples were analysed using SYBR Green qPCR Master Mix (EZBioscience, USA) and QuantStudio 3 (Appliedbiosystems, USA). Each gene was normalized to GAPDH to compare fold changes relative to control samples. Three technical replicate experiments were performed for each assay. The primers used in this study are shown in Supplementary Table S2.

### Western blotting

The total proteins were extracted by lysing cells with RIPA buffer and were then centrifuged at 12,000 g for 10 min at 4°C. Proteins in the cells were separated by SDS-PAGE and then transferred to a polyvinylidene fluoride membrane (Millipore, USA). After blocking with 5% BSA for 1 h at room temperature, the membranes were incubated overnight at 4°C with the following primary antibodies: GAPDH (ab8245, Abcam), TAGLN (10493-1-AP, Proteintech), SLC2A3 (20403-1-AP, Proteintech), COL1A1 (14695-1- AP, Proteintech), COL3A1 (22734-1-AP, Proteintech), RhoA (10749-1-AP, Proteintech), ROCK2 (21645-1-AP1, Proteintech). The next day, the membranes were incubated with anti-rabbit (7074 S, CST) or anti-mouse (7076 S, CST) IgG, HRP-linked antibody for 1 h at room temperature. The membrane was washed with 0.1% TBST and developed using iBright CL1500 (Invitrogen, USA).

### RNA sequencing

For RNA-sequencing analysis, patient-derived fibroblasts were pretreated with TAGLN-siRNA or NC-siRNA. Each experimental group contained three biological replicates. Total RNA was extracted using TRIzol reagent (Invitrogen, USA). QC qualified samples for subsequent analysis. A cDNA library was constructed using the TruSeq Stranded mRNA LT Sample Prep Kit (Illumina, USA). Analysis of differentially expressed genes was completed using the R package DEseq2 (v 1.6.3). For this study, for* P* < 0.05, a fold change > 2 or a fold change < 0.5 was set as the threshold for a significant differential expression pattern.

### Animal models

Six-week-old C57/BL6 mice were purchased from the Shanghai Slac Laboratory Animal (SLAC Experimental Animals, China). Mice were anesthetized using the R550 anesthesia machine (RWD, China) with isoflurane as the anesthetic agent specifically adapted for this equipment. For induction of anesthesia, the oxygen flow rate was set to 1 L/min and the isoflurane concentration was adjusted to 3.5%. During maintenance anesthesia via face mask, the oxygen flow rate was reduced to 0.5 L/min with an isoflurane concentration of 1%. 100 μL of PBS, AAV9-shPiezo1 or AAV9-shCtrl was injected subcutaneously into the mice 21 days prior to bleomycin injection. After 21 days, 100 μL of 0.5 mg/mL bleomycin (MedChemExpress, USA) was injected subcutaneously into the mice once a day for 4 weeks to induce dermal fibrosis. All animal procedures were performed in accordance with the guidelines of the Animal Care and Use Committee of Shanghai Jiao Tong University School of Medicine.

### Adeno-associated virus (AAV) construction and injection

Genomeditech (Shanghai, China) designed and supplied TAGLN-specific shRNA (CCAACTGGTTTATGAAGAA) and NC shRNA (TTCTCCGAACGTGTCACGT) lentiviruses. Single-stranded DNA oligonucleotides containing interfering sequences were first synthesized. These oligos were then annealed to form double-stranded DNA oligos, which were subsequently ligated into the pre-cut RNA interference vector (GM-15452) via their restriction enzyme sites at both ends. The ligation product was then transformed into competent Escherichia coli strain Stbl3 (ThermoFisher, USA). Sequencing analysis was conducted on the resulting monoclonal colonies to verify the accuracy of the cloned sequences. Clones that matched the expected design were considered to have successfully constructed the target gene RNA interference vector. Subsequently, the successfully constructed AAV expression vector and its helper packaging plasmids were extracted using a high-purity, endotoxin-free plasmid extraction kit. The viral vector and its helper packaging plasmids were then co-transfected into AAV Pro-293T cells using HG transgene reagent. After 6 to 8 hours of transfection, the medium was replaced with fresh medium supplemented with Enhancing buffer, and the cells were further incubated for 72 hours. Following cell detachment, the cell suspension rich in viral particles, along with the supernatant, was collected. The collected cell suspension underwent concentration and purification through ultracentrifugation to obtain a high-titer viral concentrate. The AAV titer was determined to be 1.56E+13 VG/mL, which was then diluted to 1E+12 vg/mL and aliquots were stored at -80°C for future use. Three weeks before bleomycin injection, 100 μL of either AAV9-shTAGLN or AAV9-shCtrl, both at a concentration of 1E+12 vg/mL, was injected subcutaneously into the dorsal skin of mice.

### Statistical analysis

Two-tailed Student's *t* test was used for comparison between two groups. One-way ANOVA was used for comparison between multiple groups.* P* values < 0.05 were considered to indicate significant differences. Results were expressed as mean ± standard deviation.

For more information about immunohistochemistry, immunofluorescence staining, cell migration assay, wound healing assay, collagen contraction assay, cell counting kit-8 assay, EDU staining, glucose consumption assays and lactate production assays, please see Supplementary Material. Primers for real-time fluorescence quantitative RT-PCR are listed in Supplementary Table S2.

## Results

### Upregulation of TAGLN expression in fibrotic skin tissues

**Figure 1:**
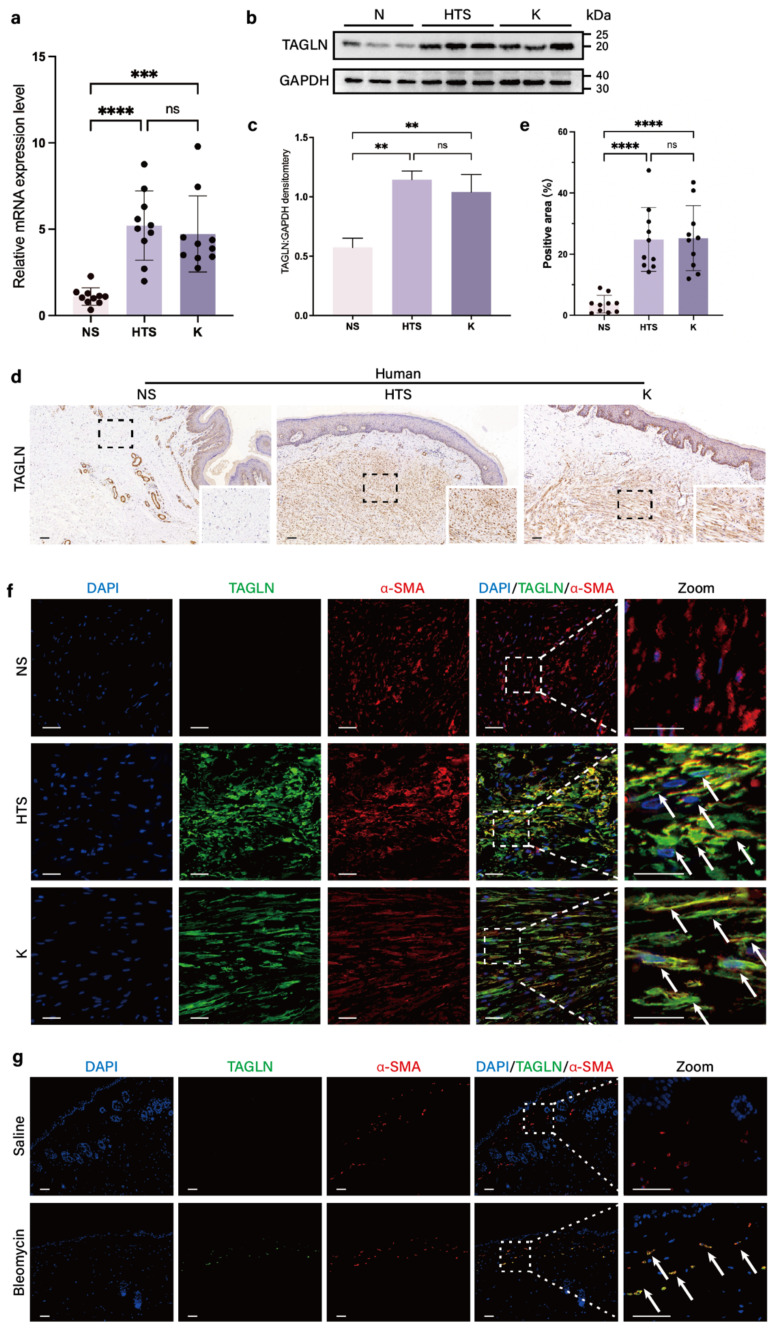
TAGLN expression is upregulated in fibrotic skin of humans and mice. (a) RT-qPCR analysis of TAGLN mRNA levels in normal skin, hypertrophic scar, and keloid fibroblasts (n = 10). (b-c) Protein levels of TAGLN were detected by western blotting. Band intensities were quantified relative to GAPDH (n = 3). (d-e) TAGLN expression in human normal skin, hypertrophic scar, and keloid, and quantitative analysis. Scale bar = 100 μm (n = 10). (f) Images of immunofluorescence co-staining of TAGLN (green) and α-SMA (red) in normal skin, hypertrophic scar, and keloid fibroblasts. Scale bar = 50 μm. (g) Images of immunofluorescence co-staining of TAGLN (green) and α-SMA (red) in mouse normal skin and skin fibrosis tissues. Scale bar = 100 μm. The results are expressed as the means ± SD. “n” means independent experiment repeat times in western blotting and independent biological samples in other analyses. Two-tailed *t* test or one-way ANOVA was used for all analyses. **P < 0.01, *** *P* < 0.005, **** *P* < 0.001.

According to our previous single-cell sequencing results (GSE243716) 37, TAGLN is a marker gene for fibroblast subpopulations enriched in human fibrotic skin tissues, including hypertrophic scars and keloids (Supplementary Figure S1a). To validate this result, we compared the expression of TAGLN in fibroblasts derived from 10 normal skin specimens, 10 hypertrophic scars, and 10 keloids by real-time quantitative PCR (RT-qPCR). There was a significant upregulation of TAGLN in pathological scar fibroblasts compared with normal skin (Figure 1a, Supplementary Figures S1b). Western blot results confirmed this finding (Figures 1b, c), and immunohistochemical analyses confirmed the increase in TAGLN in human fibrotic skin tissues (Figures 1d, e). In addition, in α-SMA/TAGLN immunofluorescence co-staining, we found that TAGLN was highly expressed in activated fibroblasts in human fibrotic skin tissues (Figure 1f). We further validated this finding in a bleomycin-induced mouse model of skin fibrosis. The effect of bleomycin-induced fibrosis was verified by hematoxylin and eosin staining and Masson staining (Supplementary Figures S1c, d). PCR and western blot confirmed that TAGLN was overexpressed in mouse fibrotic skin tissues (Supplementary Figures S1e-g). Immunohistochemical analysis showed that TAGLN was overexpressed in the fibrotic dermis of mice (Supplementary Figure S1h). Immunofluorescence co-staining with TAGLN and α-SMA showed that TAGLN was overexpressed in mouse dermal myofibroblasts Figure S1g), consistent with that observed in human fibrotic skin tissues. Taken together, these results suggest that TAGLN may be associated with skin fibrosis.

### Knockdown of TAGLN inhibits fibroblast motility *in vitro*

**Figure 2:**
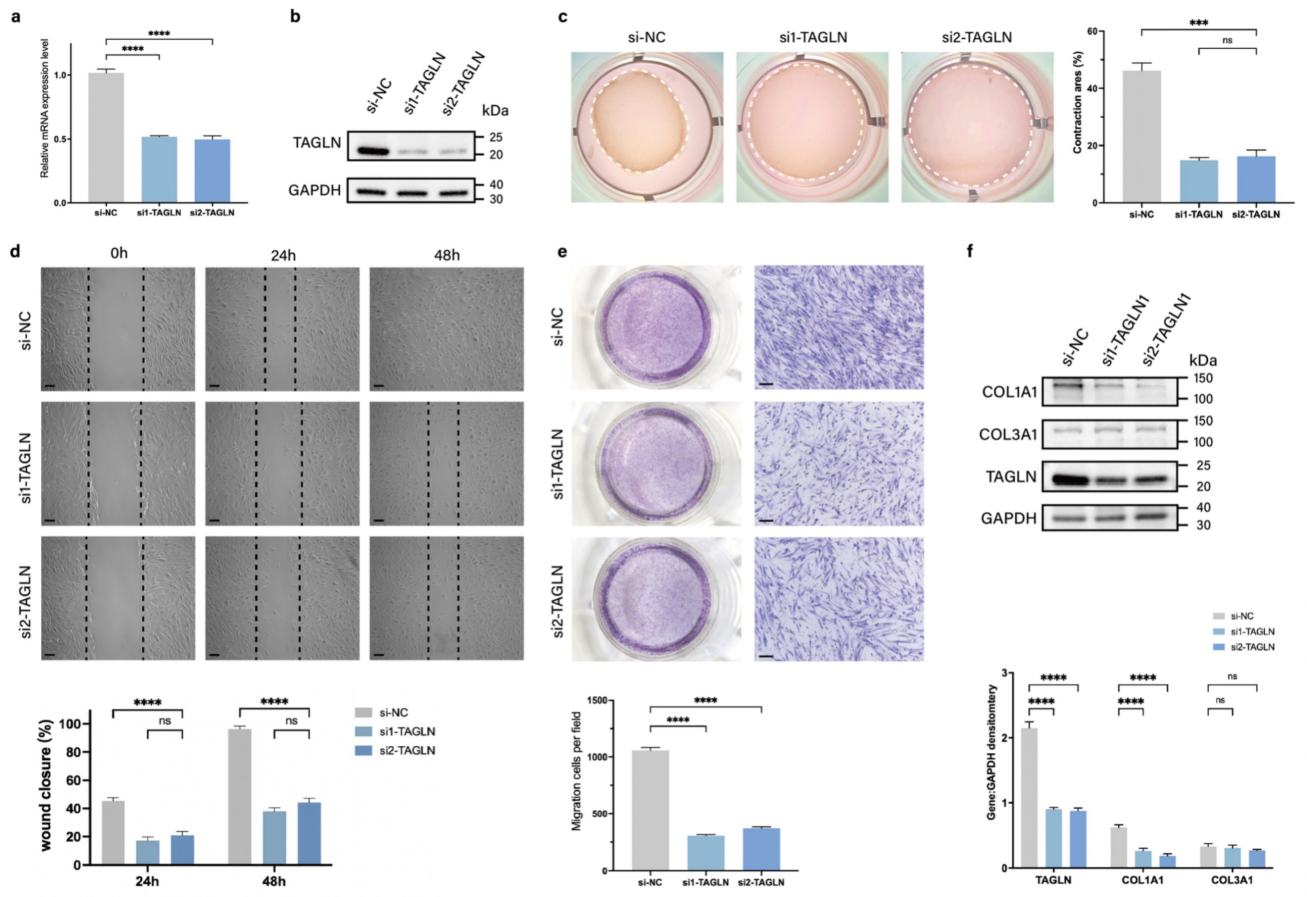
Knockdown of TAGLN inhibits the motility of patient-derived fibroblasts *in vitro*. (a-b) Identification of si-TAGLN efficiency in patient-derived fibroblasts. (c) Images and quantification of collagen gel contraction assays in different groups at 24 h after the intervention of si-TAGLN. Dashed lines indicate the areas of collagen gel. (d) Images and quantitative analysis of wound healing assays in different groups at 24 and 48 h after the intervention of si-TAGLN. Scale bar = 50 μm. (e) Images and quantitative analysis of Transwell assays of the migration of patient-derived fibroblasts in different groups at 24 h after incubation. Scale bar = 50 μm. (f) Protein levels of COL1A1 and COL3A1 were detected by western blotting. Band intensities were quantified relative to GAPDH (n = 3). The results are expressed as the means ± SD. “n” means independent experiment repeat times. Two-tailed *t* test or one-way ANOVA was used for all analyses. **P < 0.01, *** *P* < 0.005, **** *P* < 0.001.

To assess the role of TAGLN in regulating pathological scar formation, we designed two small interfering RNAs (siRNAs) targeting TAGLN (si-TAGLN) to inhibit expression of TAGLN mRNA in patient-derived fibroblasts. The knockdown efficiency was verified by qPCR and protein blotting (Figures 2a, b; Supplementary Figure S2a). We further analyzed whether TAGLN plays a potential role in the phenotype of patient-derived fibroblasts. The *in vitro* cell counting kit-8 (CCK-8) (Supplementary Figure S2b) and EdU cell proliferation assay (Supplementary Figure S2c) demonstrated no significant change in the proliferative activity of patient-derived fibroblasts after si-TAGLN treatment. However, collagen contraction assay (Figure 2c) confirmed that the contraction ability of patient-derived fibroblasts was significantly reduced after si-TAGLN treatment. The wound healing assay confirmed that the migration ability of patient-derived fibroblasts was significantly lower after si-TAGLN treatment (Figure 2d). The Transwell assay confirmed that the invasion ability of patient-derived fibroblasts was reduced after si-TAGLN treatment (Figure 2e). In addition, we found that TAGLN affects the collagen secretory activity of patient-derived fibroblasts. The expression of COL1A1 in patient-derived fibroblasts, the main secretory cell of ECM, was significantly downregulated after si-TAGLN treatment, whereas the expression of COL3A1 was not significantly changed (Figure 2f). Taken together, these results suggest that TAGLN promotes patient-derived fibroblasts motility and collagen secretory activity.

### SLC2A3 is a TAGLN-regulated downstream protein

**Figure 3:**
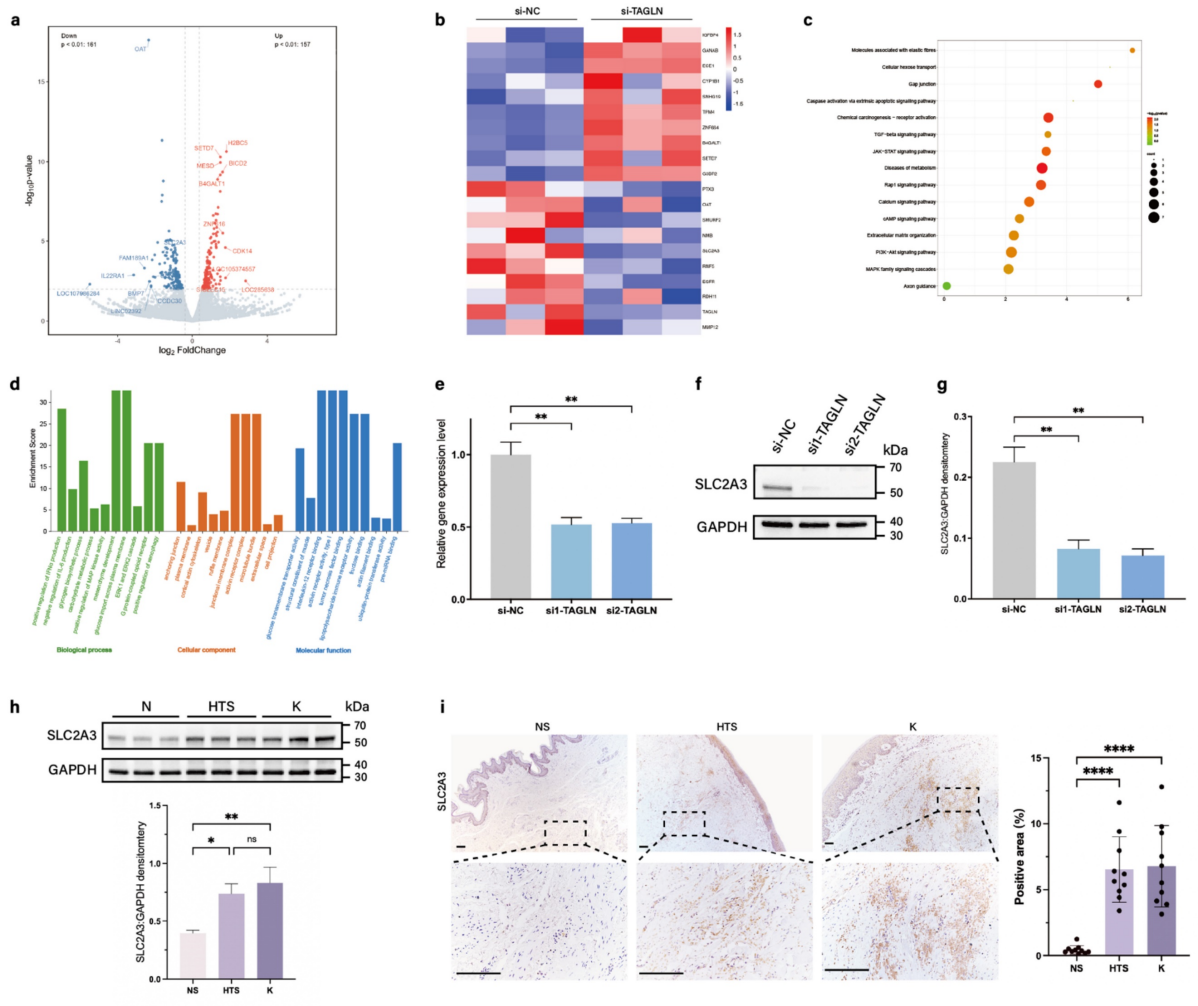
SLC2A3 is a downstream gene of TAGLN in patient-derived fibroblasts. (a) Volcano plot showing the 10 upregulated and 10 downregulated genes with significant differences in expression between si-TAGLN patient-derived fibroblasts and si-NC patient-derived fibroblasts. (b) A heat map showing hierarchical clustering of differentially expressed genes (*P* < 0.01) between si-TAGLN patient-derived fibroblasts and si-NC patient-derived fibroblasts (n = 3). (c) KEGG enrichment analysis showing the most downregulated pathways (based on RNA-sequencing data) between si-TAGLN patient-derived fibroblasts and si-NC patient-derived fibroblasts. (d) GO enrichment analysis showing the most downregulated pathways (based on RNA-sequencing data) between si-TAGLN patient-derived fibroblasts and si-NC patient-derived fibroblasts. (e) Expression of SLC2A3 was detected by PCR of mRNA expression after knockdown of TAGLN. (f-g) Expression of SLC2A3 was detected by western blotting and quantitative analysis of protein expression after knockdown of TAGLN (n = 3). (h) Protein levels of SLC2A3 in human normal skin, hypertrophic scar, and keloid fibroblasts, and quantitative analysis. Band intensities were quantified relative to GAPDH (n = 10). (i) SLC2A3 expression in human normal skin, hypertrophic scar, and keloid tissues, and quantitative analysis. Scale bar = 200 μm (n = 10). The results are expressed as the means ± SD. “n” means independent experiment repeat times in western blotting and independent biological samples in other analyses. Two-tailed *t* test or one-way ANOVA was used for all analyses. *P < 0.05, **P < 0.01, **** *P* < 0.001.

To elucidate the potential mechanism of TAGLN in skin fibrosis, we performed Bulk RNA sequencing on patient-derived fibroblasts after si-TAGLN and si-NC treatment. We first analyzed the differentially expressed genes identified in the RNA-sequencing data. A total of 178 commonly differentially expressed genes were identified based on a *P* value of ≤ 0.01, of which 157 were upregulated and 161 were downregulated (Figure 3a). Combined with the heat map (Figure 3b), we focused on one of the most significantly differentially expressed genes among the downregulated genes, SLC2A3, which encodes the glucose transporter protein 3 (GLUT3). In the KEGG enrichment analysis of downregulated pathways after si-TAGLN (Figure 3c), we found that TAGLN plays a role in regulating ECM and cell metabolism. In the GO enrichment analysis (Figure 3d), regulatory pathways associated with glucose metabolism were also highly expressed, including 'Glycogen biosynthetic process,' 'Carbohydrate metabolic process,' 'Glucose import across plasma membrane,' and 'Glucose transmembrane transporter activity.' Pathways related to fibrosis and cell motility were also highly expressed, such as 'Mesenchyme development,' 'Microtubule bundle,' and 'Extracellular space.' These data suggest that TAGLN may be an important molecule coupling cell motility and metabolism, and SLC2A3 is a key downstream of its link to cellular metabolism.

To verify whether SLC2A3 is regulated by TAGLN in patient-derived fibroblasts, we treated patient-derived fibroblasts with si-TAGLN. qPCR and western blotting showed that knockdown of TAGLN induced a decrease in SLC2A3 (Figures 3e-g). We also examined SLC2A3 expression in pathological scars and healthy skin. PCR and western blot results suggested that SLC2A3 expression was upregulated in patient-derived fibroblasts (Supplementary Figure S3a; Figure 3h). Immunohistochemical staining showed that SLC2A3 expression was significantly elevated in the dermis of pathological scars, consistent with the changes in TAGLN (Figure 3i). This finding was verified in a bleomycin-induced mouse model, where SLC2A3 was also upregulated in fibrotic skin tissues (Supplementary Figures S3b-d). Thus, the above results suggest that SLC2A3 may be a key downstream target in the regulation of fibroblast motility and metabolism by TAGLN in patient-derived fibroblasts.

### Knockdown of SLC2A3 downregulates glycolysis levels and motility of fibroblasts

**Figure 4:**
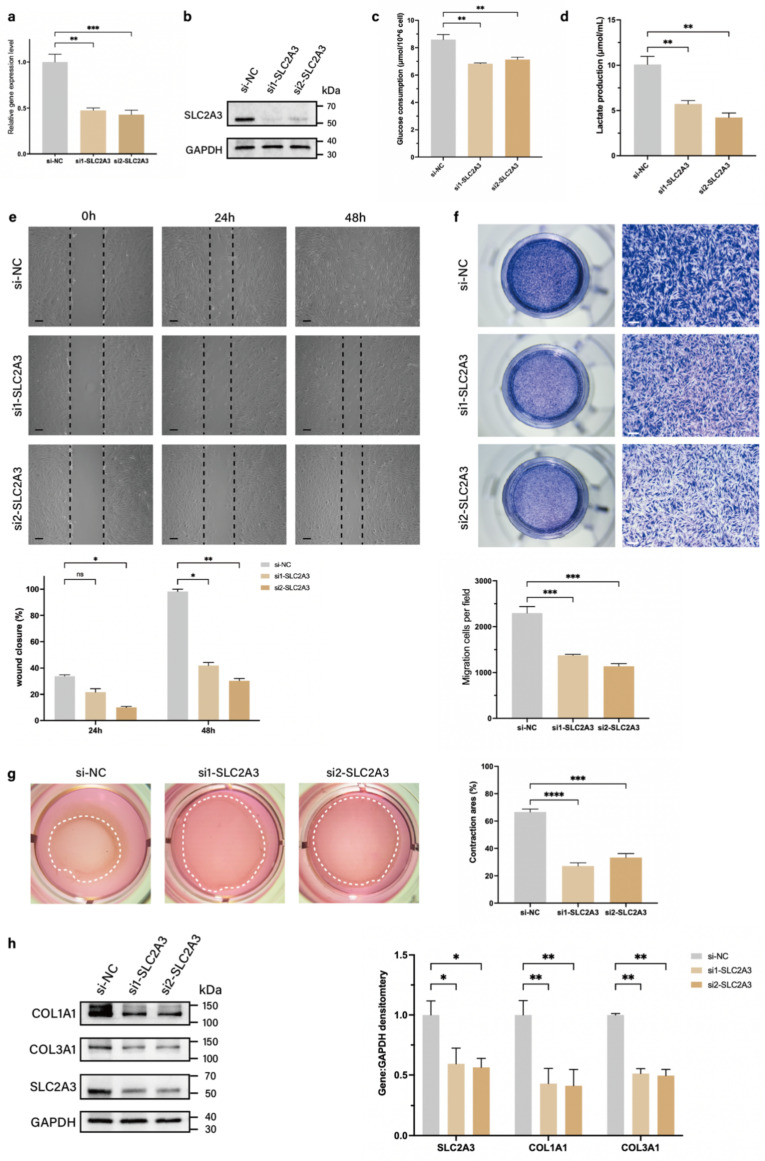
Knockdown of SLC2A3 inhibits patient-derived fibroblasts glycolysis and motility. (a-b) Identification of si-SLC2A3 efficiency in patient-derived fibroblasts. (c) Glucose consumption level in patient-derived fibroblasts was measured with a glucose assay kit. (d) Lactate production level in patient-derived fibroblasts was measured with a lactate assay kit. (e) Images and quantitative analysis of wound healing assays in different groups at 24 and 48 h after the intervention of si-SLC2A3. Scale bar = 50 μm. (f) Images and quantitative analysis of Transwell assays of the migration of patient-derived fibroblasts in different groups after incubation. Scale bar = 50 μm. (g) Images and quantification of collagen gel contraction assays in different groups at 24 h after the intervention of si-SLC2A3. Dashed lines indicate the areas of collagen gel. (h) Protein levels of COL1A1 and COL3A1 were detected by western blotting. Band intensities were quantified relative to GAPDH (n = 3). The results are expressed as the means ± SD. “n” means independent experiment repeat times. Two-tailed *t* test or one-way ANOVA was used for all analyses. *P < 0.05, **P < 0.01, *** *P* < 0.005, **** *P* < 0.001.

SLC2A3, a member of the glucose transporter protein family, is a known regulator of glycolysis (14. Therefore, we speculated that TAGLN could affect glycolysis levels and promote fibroblast motility in pathological scarring by regulating SLC2A3. We designed two small interfering RNAs (siRNAs) targeting SLC2A3 (si-SLC2A3) to inhibit the expression of SLC2A3 mRNA in patient-derived fibroblasts, and verified the knockdown efficiencies by qPCR and protein blotting (Figures 4a, b; Supplementary Figure S4a). We further examined the glycolysis levels and phenotypic changes in patient-derived fibroblasts after si-SLC2A3 treatment. The results showed that after knockdown of SLC2A3, the levels of glucose uptake and lactate production in patient-derived fibroblasts were significantly reduced (Figures 4c, d), suggesting that the glycolytic process was inhibited. CCK-8 (Supplementary Figure S4b) and EdU cell proliferation assay (Supplementary Figure S4c) suggested that the inhibition of SLC2A3 did not significantly alter the proliferative activity of patient-derived fibroblasts. The wound healing assay (Figure 4e) and Transwell assay (Figure 4f) confirmed that the migration and invasion abilities of patient-derived fibroblasts were significantly downregulated after si-SLC2A3 treatment. The collagen contraction assay confirmed that the contraction ability of collagen was significantly reduced after si-SLC2A3 treatment (Figure 4g). In addition, we found that SLC2A3 affected the secretory activity of patient-derived fibroblasts. Both COL1A1 and COL3A1 expression levels of patient-derived fibroblasts were significantly downregulated after si-SLC2A3 treatment (Figure 2h). Taken together, these results suggest that SLC2A3 regulates the glycolysis level of patient-derived fibroblasts and enhances their motility and secretory function.

### TAGLN activates SLC2A3 through the RhoA/ROCK2 pathway

**Figure 5:**
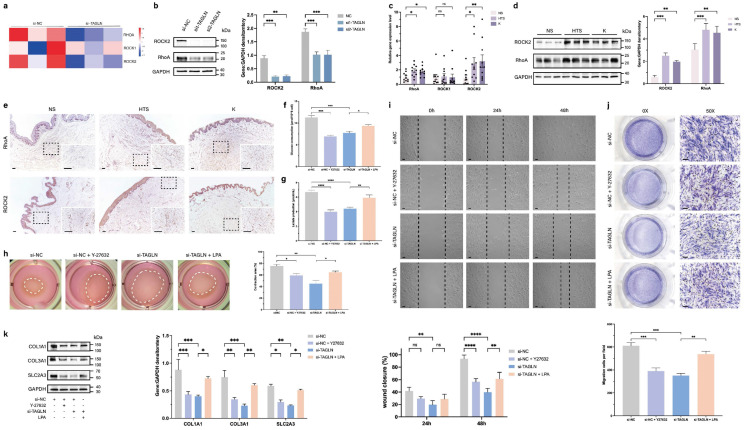
TAGLN activates SLC2A3 through the RhoA/ROCK2 pathway. A heat map showing hierarchical clustering of gene expression level of RhoA, ROCK1, and ROCK2 between si-TAGLN patient-derived fibroblasts and si-NC patient-derived fibroblasts (n = 3). (b) Protein levels of RhoA and ROCK2 of different groups were detected by western blotting. Band intensities were quantified relative to GAPDH (n = 3). (c) RT-qPCR analysis of RhoA, ROCK1, and ROCK2 mRNA levels in normal skin, hypertrophic scar, and keloid fibroblasts (n = 10). (d) Protein levels of RhoA and ROCK2 were detected by western blotting. Band intensities were quantified relative to GAPDH (n = 3). (e) RhoA and ROCK2 expression in human normal skin, hypertrophic scar, and keloid tissues. Scale bar = 100 μm. (f) Glucose consumption level in patient-derived fibroblasts was measured with a glucose assay kit. (g) Lactate production level in patient-derived fibroblasts was measured with a lactate assay kit. (h) Images and quantification of collagen gel contraction assays in different groups at 24 h after different interventions. Dashed lines indicate the areas of collagen gel. (i) Images and quantitative analysis of wound healing assays in different groups at 24 and 48 h after different interventions. Scale bar = 50 μm. (j) Images and quantitative analysis of Transwell assays of the migration of patient-derived fibroblasts in different groups at 24 h after incubation. Scale bar = 50 μm. (k) Protein levels of COL1A1, COL3A1, and SLC2A3 were detected by western blotting. Band intensities were quantified relative to GAPDH (n = 3). The results are expressed as the means ± SD. “n” means independent experiment repeat times. Two-tailed *t* test or one-way ANOVA was used for all analyses. *P < 0.05, **P < 0.01, *** *P* < 0.005, **** *P* < 0.001.

TAGLN has been identified as an important mechanosensitive gene. In ovarian cancer, TAGLN senses changes in environmental stiffness and mediates ovarian cancer progression by regulating the RhoA/ROCK pathway 11. RhoA and ROCK are important molecules that mediate mechanotransduction 15,16. Therefore, we explored whether TAGLN in patient-derived fibroblasts activates SLC2A3 through the RhoA/ROCK pathway by examining the expression of RhoA and ROCK in the RNA-sequencing data. We found that RhoA and ROCK2 were downregulated in the si-TAGLN group relative to the si-NC group (Figure 5a). To further validate this observation, we knocked out TAGLN in patient-derived fibroblasts and reached the same conclusion through western blotting (Figure 5b). We then compared the expression of RhoA and ROCK2 in fibroblasts from 10 normal skin samples, 10 hypertrophic scars, and 10 keloids by qPCR and western blot (Figure 5c, d). There was a significant upregulation of RhoA and ROCK2 in fibroblasts from pathological scars compared with those from normal skin. Immunohistochemical analysis confirmed the increased expression of RhoA and ROCK2 in human pathological scar tissue (Figure 5e; Supplementary Figures S5a, b).

To investigate the effect of RhoA/ROCK2 in patient-derived fibroblasts and whether its overexpression could rescue the phenotypic changes caused by TAGLN, we exogenously added agonist (lysophosphatidic acid, LPA) and inhibitor (Y-27632) of the RhoA/ROCK2 pathway (Supplementary Figures S5c) 17-20. The results showed that glucose uptake and lactate production in patient-derived fibroblasts were significantly reduced after exogenous addition of Y-27632, suggesting that the level of glycolysis was inhibited. The inhibition of glycolysis after TAGLN knockdown was rescued by the addition of LPA (Figures 5f, g). After exogenous addition of Y-27632, cell motility and secretory ability were also inhibited, including cell contractility (Figure 5h), migration (Figure 5i), invasion (Figure 5j), and synthesis of ECM components (Figure 5k). Exogenous administration of LPA rescued the inhibition of glycolytic levels, motility, and secretory capacity of patient-derived fibroblasts by TAGLN knockdown. Collectively, these data suggest that TAGLN can regulate SLC2A3 expression through the RhoA/ROCK2 pathway. Therefore, the TAGLN-RhoA/ROCK2- -SLC2A3 pathway is required for TAGLN-mediated pathological scar progression.

### AAV9-targeted knockdown of TAGLN attenuates skin fibrosis in a bleomycin-induced mouse model

**Figure 6:**
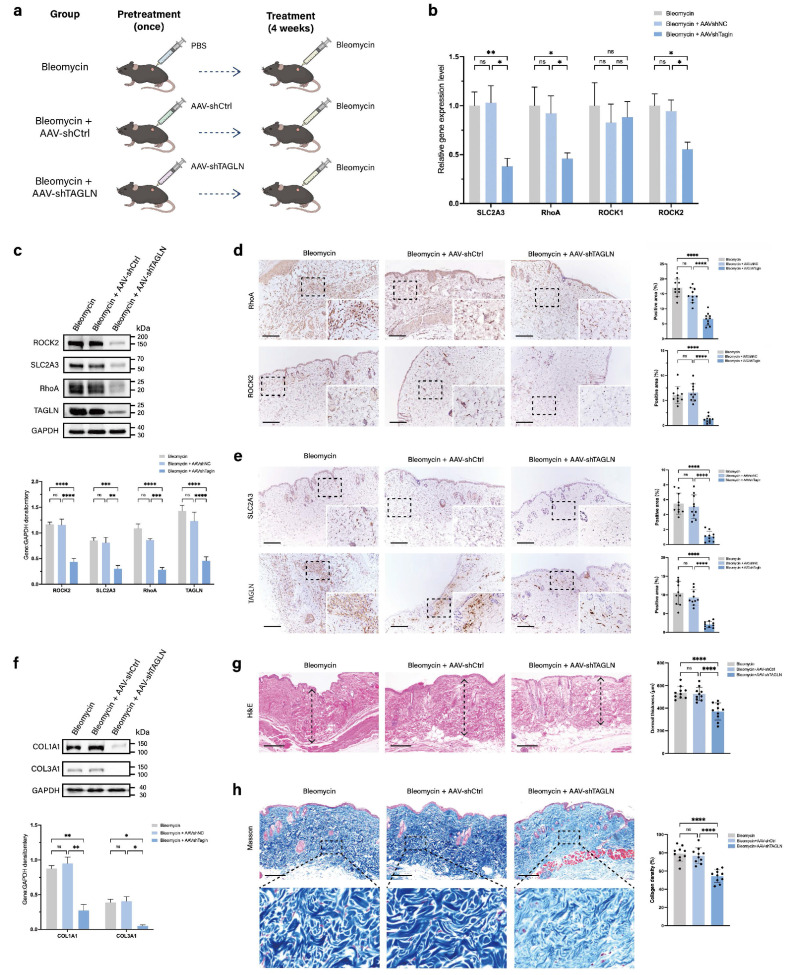
Targeted knockdown of Tagln with AAV9 attenuates the progression of skin fibrosis in mice. (a) Schematic representation of the construction of the mouse fibrosis model and experimental methods. The mice were divided into three groups and received pretreatment with PBS, AAV9-shCtrl, or AAV9-TAGLN. Then, all of the groups received 4 weeks of bleomycin injection to induce skin fibrosis. (b) mRNA levels of SLC2A3, RhoA, ROCK1, and ROCK2 in skin tissues from the three groups of mice (n = 10). (c) Protein levels and quantitative analysis of TAGLN, SLC2A3, RhoA, and ROCK2 in skin tissues of mice from the three groups (n = 3). (d-e) Images and quantitative analysis of immunohistochemical staining of RhoA, ROCK2, SLC2A3, and TAGLN in the three groups. Scale bar = 200 μm (n = 10). (f) Protein levels and quantitative analysis of COL1A1 and COL3A1 in skin tissues of mice from the three groups (n = 3). (g) Representative images of hematoxylin and eosin staining, and quantitative analysis of the results of the three groups. The height of the arrow indicates the thickness of the dermis. Scale bar = 200 μm (n = 10). (h) Representative images and quantitative analysis of collagen deposition shown by Masson staining of the three groups. Scale bar = 200 μm (n = 10). The results are expressed as the means ± SD. “n” means independent experiment repeat times in western blotting and independent biological samples in other analyses. One-way ANOVA was used for all analyses. *P < 0.05, **P < 0.01, *** *P* < 0.005, **** *P* < 0.001.

To further validate whether targeting TAGLN modulates pathological scar progression, we investigated the effects of AAV9-shTAGLN treatment in a bleomycin-induced mouse model of skin fibrosis. We injected PBS, AAV9-shTAGLN, or AAV9-shCtrl into the dorsal skin of the mice. After 3 weeks, TAGLN expression was significantly reduced in AAV9-shRNA-treated mice compared with PBS- or AAV9-shCtrl-treated mice (Supplementary Figure S6a, b). Subsequently, we induced dermal fibrosis in the mice by continuous 4-week bleomycin injection (Figure 6a). We examined the levels of TAGLN downstream signaling pathways and molecules, including SLC2A3, RhoA, and ROCK2, in three groups, namely the bleomycin group, the AAV9-shCtrl group, and the AAV9-shTAGLN group. Specifically, we performed western blot and immunohistochemical validation of the tissues. The results showed that TAGLN knockdown downregulated the expression of SLC2A3, RhoA, and ROCK2 *in vivo* (Figures 6c-e) and decreased collagen secretion (Figure 6f). The degree of bleomycin-induced fibrosis was relatively mild in the AAV9-shTAGLN-treated group, with reduced dermal thickness and collagen deposition compared with the AAV9-shCtrl-treated group (Figures 6g-h). Taken together, our data confirm the role of the TAGLN-RhoA/ROCK2-SLC2A3 pathway in skin fibrosis.

## Discussion

Mechanical tension has been reported to be one of the key cues regulating skin fibrosis 21,22. Increased local mechanical tension in the wound stimulates capillary growth, induces fibroblast proliferation and activation, and promotes the progression of fibrosis in scarring as a result 23. Furthermore, recent studies have reported the presence of sustained enhanced aerobic glycolysis in fibrotic skin diseases, including hypertrophic scars and keloids. Imbalanced energy metabolism during tissue remodeling leads to fibroblast proliferation, activation, and excessive accumulation of ECM 7,24-26. However, it has not been reported whether there is an association between mechanical tension and glucose metabolism during the development of skin fibrosis. In the present study, we found that the expression of the cytoskeletal protein TAGLN was upregulated in skin fibrotic tissues, and the coupling of mechanomechanics and glucose metabolism was mediated through the TAGLN-RhoA/ROCK2-SLC2A3 axis, which drives progression of skin fibrosis.

Through the results of preliminary single-cell sequencing, we identified an enriched subpopulation of fibroblasts in fibrotic skin tissues that highly express TAGLN. TAGLN encodes actin cross-linking proteins, which act as the glue of the cytoskeleton. As a mechanosensitive gene, TAGLN can sense mechanical force changes and plays a key role in cell motility and mechanosensing 11. It has been shown that TAGLN-positive myofibroblasts have an enhanced ability to proliferate, adhere, and migrate *in vitro* 27. In this study, we demonstrated that TAGLN expression was upregulated in myofibroblasts from human pathological scars and mouse fibrotic skin tissue. By knocking down the expression of TAGLN, we found that downregulation of TAGLN inhibited the motility and secretory functions of patient-derived fibroblasts, including invasion, migration, contraction, and collagen secretion. However, unlike previous studies in which patient-derived fibroblasts showed hyperproliferation (Li *et al.* 2016; Zhang *et al.* 2023), in our study TAGLN did not affect the proliferative activity of fibroblasts. Therefore, we speculated that TAGLN influences the development of pathological scarring by a novel mechanism.

SLC2A3 has the highest affinity for glucose among the family of glucose transporter proteins. Thus, it plays a crucial role in providing energetic fuel to a wide range of cells, especially those requiring high level of glucose and having the Warburg effect, such as neurons, cardiomyocytes, and tumor cells 28-30. In single-cell metabolic imaging of endothelial cells, it has been observed that SLC2A3 acts as a transporter protein that mediates the glycolytic burst and enhances cell motility 31. Our study demonstrated that downregulation of TAGLN inhibited SLC2A3 expression, which reduced cellular glycolysis levels and cellular motility. This suggests that mechanotransduced cytoskeletal proteins can regulate glycolytic processes in skin fibrosis.

Interestingly, the differential effects of TAGLN and SLC2A3 knockdown on collagen expression were observed in our study. While TAGLN knockdown in fibroblasts specifically reduced COL1A1 secretion without affecting COL3A1, the knockdown of SLC2A3 in the same cell type led to a reduction in both collagen types. However, the knockdown of TAGLN in mouse skin using AAV resulted in a significant reduction in both COL1A1 and COL3A1. These findings suggest that TAGLN and SLC2A3 may not operate within a single, exclusive pathway regulating collagen expression. Furthermore, the involvement of other genes and pathways, as well as the influence of different cell types within the skin tissue, could contribute to the observed differences in collagen expression.

The Rho/ROCK signaling pathway is one of the most widely affected pathways in cytoskeletal dynamics, whose function is to regulate the assembly of actin filaments and actin-globin contraction 32,33. The Rho/ROCK signaling pathway promotes stress fiber formation and scar contraction and plays an important role in many fibrotic diseases 34-36. Previous studies have shown that RhoA/ROCK-mediated coupling of glycolysis and cell contraction is a key pathway for the close coordination of cellular metabolism and cytoskeletal structure in 'high-energy metabolism' 31. Therefore, we focused on the downregulation of the RhoA/ROCK pathway after TAGLN knockdown. We further investigated whether the RhoA/ROCK pathway connects the mechanosensing to metabolic motility of patient-derived fibroblasts and regulates it in the fibrotic progression. The results showed that TAGLN regulated the expression of SLC2A3 through the RhoA/ROCK2 pathway, which in turn promoted glycolysis and cell motility in patient-derived fibroblasts. In addition, we further performed *in vivo* experiments to explore the therapeutic potential of TAGLN in fibrotic diseases. The results showed that AAV-targeted TAGLN knockdown effectively ameliorated the progression of skin fibrosis in mice.

This study also has some limitations. Firstly, the lack of investigation into the overexpression of TAGLN has hindered our ability to comprehensively explore the regulatory effects of TAGLN on downstream gene expression. Secondly, the specific molecular mechanisms how TAGLN senses the changes in mechanical forces and activates RhoA/ROCK2 have not been investigated in pathological scarring. Moreover, it remains unclear how RhoA/ROCK2 regulates the transmembrane expression of SLC2A3. These aspects will be the focus for further refinement and exploration in future studies.

## Conclusion

This study identified a mechano-metabolic axis in the progression of skin fibrosis, namely the TAGLN-RhoA/ROCK2-SLC2A3 axis. In fibroblasts, TAGLN can regulate the level of SLC2A3-mediated glycolysis and motility through the RhoA/ROCK2 pathway, thereby promoting fibrotic progression. This study provides a framework for better understanding the effects of mechanical environment on cellular metabolic alterations and cell motility. Thus, therapies targeting the TAGLN-RhoA/ROCK2-SLC2A3 axis could break the coupling between cellular mechanotransduction and aberrant cellular metabolism. This holds great promise as a therapeutic target for skin fibrosis and other fibrotic diseases.

## Supplementary Material

Supplementary methods, figures and tables.

## Abbreviations

ECM: extracellular matrix
Transgelin: TAGLN
DMEM: Dulbecco's modified eagle medium
siRNAs: small interfering RNAs
NC: negative control
AAV: adeno-associated virus
EDU: 5-Ethynyl-2′-deoxyuridine
CCK-8: cell counting kit-8
qPCR: quantitative polymerase chain reaction
KEGG: Kyoto Encyclopedia of Genes and Genomes‌
GO: Gene Ontology
LPA: lysophosphatidic acid
shRNAs: short hairpin RNAs
