# Supplementary material for: TAGLN-RhoA/ROCK2-SLC2A3-mediated Mechano-metabolic Axis Promotes Skin Fibrosis
Source: Int J Biol Sci. 2025 Jan 1;21(2):658–70. doi: 10.7150/ijbs.104484 (PMC11705643; doi:10.7150/ijbs.104484)

## SUPPORTING INFORMATION

### 1. Supplementary Table 1. Patient information

| Order | Sex | Age (years) | Location | Usage |
|---|---|---|---|---|
| Normal skin-1 | Male | 6 | Foreskin | PCR and WB |
| Normal skin-2 | Male | 9 | Foreskin | PCR and WB |
| Normal skin-3 | Male | 12 | Foreskin | PCR, WB and IHC |
| Normal skin-4 | Male | 23 | Face | PCR, WB and IHC |
| Normal skin-5 | Male | 14 | Foreskin | PCR, WB and IHC |
| Normal skin-6 | Male | 10 | Foreskin | PCR, WB and IHC |
| Normal skin-7 | Female | 27 | Abdomen | PCR, WB and IHC |
| Normal skin-8 | Male | 16 | Foreskin | PCR, WB and IHC |
| Normal skin-9 | Male | 11 | Foreskin | PCR and IHC |
| Normal skin-10 | Male | 34 | Foreskin | PCR, WB, IHC and IF |
| Normal skin-11 | Male | 7 | Foreskin | IHC and IF |
| Normal skin-12 | Female | 18 | Face | IHC and IF |
| Hypertrophic scar-1 | Male | 19 | Face | PCR, WB and IHC |
| Hypertrophic scar-2 | Male | 42 | Arm | PCR, WB and IHC |
| Hypertrophic scar-3 | Male | 28 | Neck | PCR, WB and IHC |
| Hypertrophic scar-4 | Female | 31 | Abdomen | PCR, WB and IHC |
| Hypertrophic scar-5 | Female | 34 | Abdomen | PCR, WB and IHC |
| Hypertrophic scar-6 | Male | 28 | Neck | PCR, WB and IHC |
| Hypertrophic scar-7 | Female | 21 | Face | PCR and WB |
| Hypertrophic scar-8 | Female | 27 | Arm | PCR, WB and IHC |
| Hypertrophic scar-9 | Male | 42 | Chest | PCR and IHC |
| Hypertrophic scar-10 | Female | 23 | Face | PCR , WB and cytological experiments |
| Hypertrophic scar-11 | Male | 33 | Chest | Cytological experiments |
| Hypertrophic scar-12 | Female | 29 | Abdomen | IF and cytological experiments |
| Hypertrophic scar-13 | Female | 36 | Chest | PCR, WB and IF |
| Hypertrophic scar-14 | Male | 29 | Face | IHC, IF and cytological experiments |
| Hypertrophic scar-15 | Female | 30 | Abdomen | IHC and IF |
| Keloid-1 | Male | 25 | Dorsum | PCR, WB and IHC |
| Keloid-2 | Male | 28 | Chest | PCR, WB and IHC |
| Keloid-3 | Female | 22 | Chest | PCR, WB and IHC |
| Keloid-4 | Male | 50 | Dorsum | PCR, WB and IHC |
| Keloid-5 | Female | 32 | Arm | PCR, WB and IHC |
| Keloid-6 | Female | 35 | Abdomen | PCR, WB, IHC and IF |
| Keloid-7 | Male | 27 | Chest | PCR and WB |
| Keloid-8 | Female | 30 | Dorsum | PCR and WB |
| Keloid-9 | Male | 47 | Chest | PCR, IHC and IF |

| | | | | |
|---|---|---|---|---|
| Keloid-10 | Male | 36 | Chest | PCR, WB, IHC and IF |
| Keloid-11 | Female | 29 | Abdomen | sc-RNAseq and cytological experiments, IHC and and cytological experiments |
| Keloid-12 | Male | 26 | Neck | sc-RNAseq and cytological experiments, IHC and and cytological experiments |
| Keloid-13 | Female | 21 | Earlobe | sc-RNAseq and cytological experiments, IHC and IF |
| Keloid-14 | Male | 29 | Chest | Cytological experiments |
| Keloid-15 | Female | 24 | Earlobe | PCR, WB and and cytological experiments |

## 2. Supplementary Table 2. Primer list

| **Human Gene** | **Forward Primer 5’→3’** | **Reverse Primer 5’→3’** |
|---|---|---|
| TAGLN | TGTCCGAACCCAGACACAAG | CAGCCAATGCACTCACAAGG |
| SLC2A3 | GTCACTTTGCTCTGGGTGGA | AATGGGACCCTGCCTTACTG |
| RhoA | GAGCCGGTGAAACCTGAAGA | CCCCAGAGCTATGCCAACAA |
| ROCK1 | GGTTTTGTTCGTGCTTCCCC | CACAGGGCACTCAGTCACAT |
| ROCK2 | CCCCAAAAGGAGAAGACCCC | AATCCGGTGTTCCAACTGCT |
| GAPDH | ACAACTTTGGTATCGTGGAAGG | GCCATCACGCCACAGTTTC |
| **Mouse Gene** | **Forward Primer 5’→3’** | **Reverse Primer 5’→3’** |
| TAGLN | GAGCCTGTGTGGAGTGAGTG | GGCTGTCTGTGAAGTCCCTC |
| SLC2A3 | TCTCCTAAGTCACCGAGCCA | CTGAACAGCAAACCCAAGCC |
| RhoA | AAGACAGTGAGGGTTTGGGTG | CTGCGTTCACAAGGCTTCAC |
| ROCK1 | TGAAAGCCGCACTGATGGAT | TGCCATCTATTCATTCCAGCCA |
| ROCK2 | AAACTGTGATCCCAAGGGAAGG | CAAAGTGAATCGGAGGCGGA |
| GAPDH | CCCGTAGACAAAATGGTGAA | TGCCGTGAGTGGAGTCATAC |

## 3. Supplementary Methods

### Histology and immunohistochemistry

Tissues were fixed in 4% paraformaldehyde overnight, then embedded in paraffin and cut into 5 mm slices. Sections were stained with hematoxylin and eosin (H&E) or

Masson's trichrome according to the manufacturer's protocol. To perform immunohistochemical staining, the following primary antibodies were used: TAGLN (10493-1-AP, Proteintech), SLC2A3 (20403-1-AP, Proteintech), RhoA (10749-1-AP, Proteintech), ROCK2 (21645-1-AP1, Proteintech). Images were captured using a Zeiss axio vert A1 microscope (Zeiss, USA). Images were quantitatively analyzed using ImageJ software.

**Immunofluorescence staining**

Tissues were fixed with 4% PFA overnight at room temperature. Tissue samples were embedded in paraffin and cut into 5mm slices. Samples were incubated overnight at 4°C with the following antibodies: TAGLN (10493-1-AP, Proteintech), SLC2A3 (20403-1-AP, Proteintech), RhoA (10749-1-AP, Proteintech), ROCK2 (21645-1-AP1, Proteintech), α-SMA, and α-SMA. Proteintech), α-SMA (ab7817, Abcam). The corresponding secondary antibodies were then incubated. Fluorescence was analysed and images were taken using a LEICA Stellaris 8 laser confocal microscope (LEICA, Germany).

**Migration assays**

Invasion experiments were performed using chambers consisting of Transwell membrane filter inserts (Corning Costar, USA). Briefly, 5 × 10^4 cells were inoculated into each 24-well Transwell chamber (8 μm pore size) with 200 μl of serum-free medium. Complete medium containing 20% FBS was added to the lower

chamber. The invasion cycle was 24 h or 48 h. Cells that did not penetrate the filters were wiped out and cells on the lower surface of the filters were stained with 0.4% crystal violet. The number of infiltrating cells was counted from five fields of view in a single chamber (mean ± SD) of three samples under a light microscope.

**Wound healing assay**

Inoculate HPSF in 6-well plates until cell fusion is greater than 90%. Linear defects were created on the cells using a sterilised pipette tip. Cells were grown in serum-free DMEM supplemented with 1% penicillin/streptomycin. To quantify cell migration, each group measured the wound width in 3 randomly selected areas at each time point. The migration distance was measured as the difference in width between the immediate, 24, and 48 hours after wound healing. The migration distance for each sample was normalized to the ratio to the initial wound width.

**Cell Contraction Assay**

HPSFs were resuspended in 4mg/mL Collagen I Rat Tail (Corning, USA). The suspension was spread in each well of a 24-well plate. The plates were incubated at 37˚C for 30 min. After the gel had solidified, the gel was released with a syringe needle and 1 ml of DMEM supplemented with 10% FBS was added to each well. the well plates were placed in a cell incubator for 12 h before gel images were taken.

**Cell Counting Kit-8 (CCK-8) Assay**

Different groups of HPSFs were treated and inoculated into 96-well plates (3000 cells per well). At 0 h, 24 h, 48 h, 72 h and 96 h after seeding, 10 µl of CCK-8 solution (Beyotime Biotechnology, China) was added to each well. Cells were then incubated at 37 °C for 2 h for cell proliferation assay.

**5-ethynyl-20-deoxyuridine (EdU) staining**

Different groups of HPSFs were treated and inoculated in 48-well plates and incubated under standard conditions. EdU staining was detected using the Click-iT EdU Imaging Kit (Invitrogen, USA). Cells were incubated with 50 mM EdU for 2 h prior to fixation, permeabilisation and EdU staining according to the manufacturer's experimental protocol. Cell nuclei were stained with DAPI (Sigma-Aldrich, USA) at a concentration of 1 µg/ mL.

**Glucose consumption and lactate production**

Glucose consumption and lactate production were measured using a Glucose Assay Kit (Solarbio, China) and Lactate Assay Kit (Solarbio, China), respectively. 10x10^7 cells were collected from each group and assayed for glucose uptake and lactate accumulation according to the production instructions. Absorbance at 505 nm and 570 nm was recorded using an enzyme labeler (Thermo Fisher Scientific, USA) to detect glucose consumption and lactate production.

**Adeno-associated virus (AAV) construction and injection**

Genomeditech (Shanghai, China) designed and supplied TAGLN-specific shRNA (CCAACTGGTTTATGAAGAA) and NC shRNA ( TTCTCCGAACGTGTCACGT) lentiviruses. Three weeks before bleomycin injection, 100 μL (1E+12vg/ml) of AAV9-shTAGLN or AAV9-shCtrl was injected subcutaneously into the dorsal skin of mice.

## 4. Supplementary Figure legends

**Supplementary Figure S1**

(a) UMAP plot showing the distribution of TAGLN in fibroblasts in pathological scarring. (b) Images of immunofluorescence staining of Vimentin (green) in patient-derived fibroblasts. Scale bar = 50 μm. (c) Representative images of hematoxylin and eosin staining, and quantitative analysis of the results of the two groups. The height of the arrow indicates the thickness of the dermis. Scale bar = 200 μm (n = 5). (d) Representative images and quantitative analysis of collagen deposition shown by Masson staining of the two groups. Scale bar = 200 μm (n = 5). (e) RT–qPCR analysis of TAGLN mRNA levels in normal skin, hypertrophic scar and keloid fibroblasts (n = 10). (f, g) Protein levels of TAGLN were detected by Western Blotting. Band intensities were quantified relative to GAPDH. (h) TAGLN expression in mouse normal skin, skin fibrosis tissues, and quantitative analysis (n = 10). Scale bar = 100 μm. The results are expressed as the means ± SD. “n” meaning biologically independent animals. Two-tailed t-test is used for all analyses. **P < 0.01, ***P < 0.005.

**Supplementary Figure S2**

(a) The quantitative analysis of protein levels of TAGLN detected by Western Blotting in si-TAGLN HPSFs and si-NC HPSFs. Band intensities were quantified relative to GAPDH (n = 3). (b) Cell Counting Kit-8 shows cell proliferation rate of HPSFs in different groups. (c) Images of EdU proliferation assay for cultured HPSFs after transfection of si-TAGLN and si-NC plasmid for 72h. Scale bar = 50 μm. ****P < 0.001.

**Supplementary Figure S3**

(a) RT–qPCR analysis of SLC2A3 mRNA levels in normal skin, hypertrophic scar and keloid fibroblasts (n = 11). (b) RT–qPCR analysis of SLC2A3 mRNA levels in mouse normal skin, skin fibrosis tissues (n = 5). (c) SLC2A3 protein levels in mouse fibroblasts from normal skin and skin fibrosis tissues were detected by Western Blotting. Band intensities were quantified relative to GAPDH. (d) SLC2A3 expression in mouse normal skin, skin fibrosis tissues, and quantitative analysis. Scale bar = 100 μm (n = 10). *P < 0.05, **P < 0.01.

**Supplementary Figure S4**

(a) The quantitative analysis of protein levels of SLC2A3 detected by Western Blotting in si-SLC2A3 HPSFs and si-NC HPSFs. Band intensities were quantified relative to GAPDH (n = 3). (b) CCK8 shows cell proliferation rate of HPSFs in different groups. (c) Images and quantitative analysis of EdU proliferation assay for

cultured HPSFs after transfection of si-SLC2A3 and si-NC plasmid for 72h. Scale bar = 50 µm. ****P < 0.001.

**Supplementary Figure S5**

(a–b) Quantitative analysis of immunohistochemical staining of RhoA, ROCK2 and ROCK2 in normal skin, hypertrophic scar and keloid fibroblasts (n =5). (c) RT-qPCR analysis of RhoA and ROCK2 mRNA levels in fibroblasts from different treatment groups (n = 5). *P < 0.05, **P < 0.01, ***P < 0.005, ****P < 0.001.

**Supplementary Figure S6**

(a) RT–qPCR analysis of TAGLN mRNA levels in AAV-shNC pre-treated and AAV-shTAGLN pre-treated groups (n =10). (b) Images and quantitative analysis of immunohistochemical staining of TAGLN in two groups. Scale bar: 100 µm (n = 5). The results are expressed as the means ± SD. "n" meaning biologically independent mice. Two-tailed t-test is used for all analyses. *P < 0.05, **P < 0.01.

## 5. Supplementary Figures

### Supplementary Figure S1

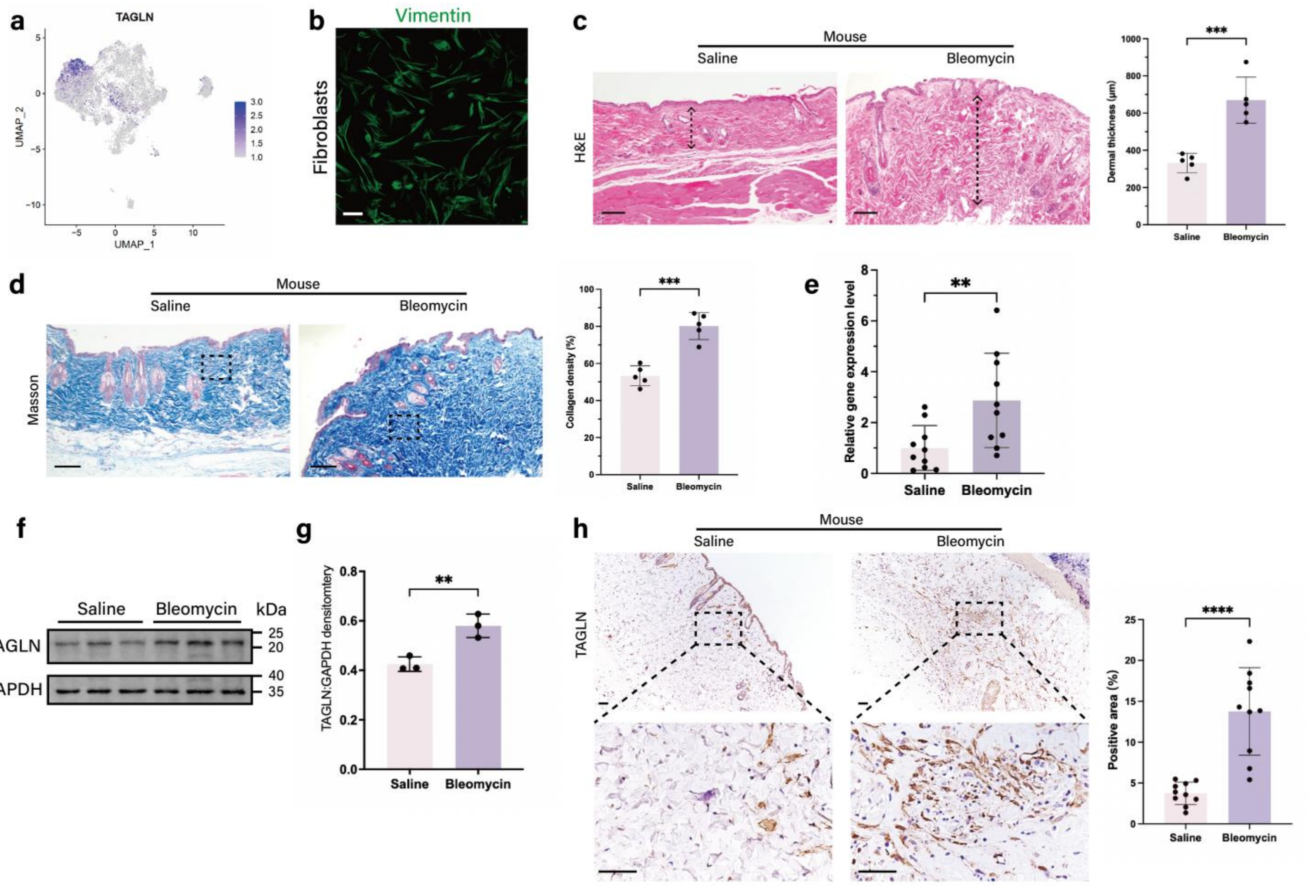


### Supplementary Figure S2

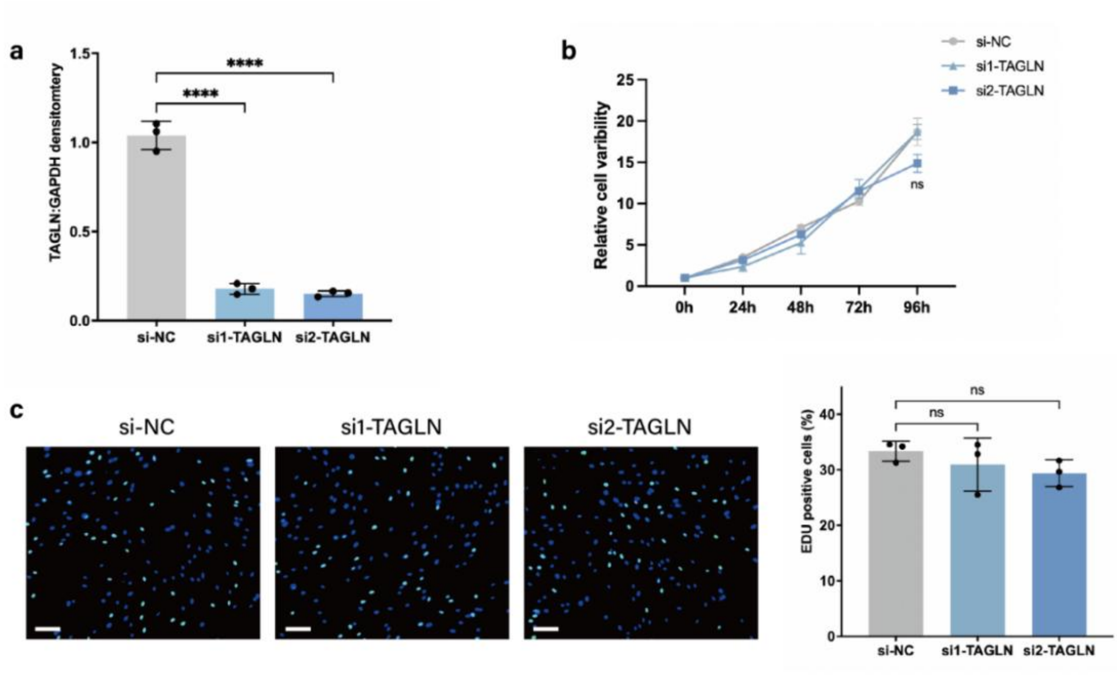

## Supplementary Figure S3

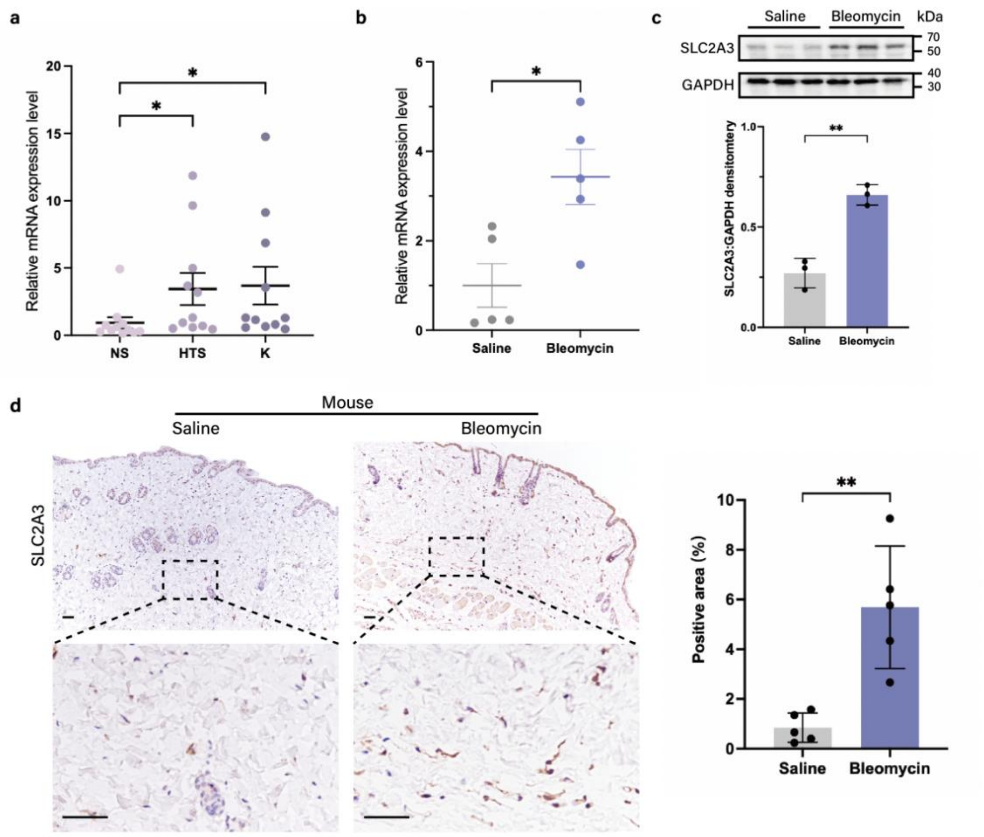


## Supplementary Figure S4

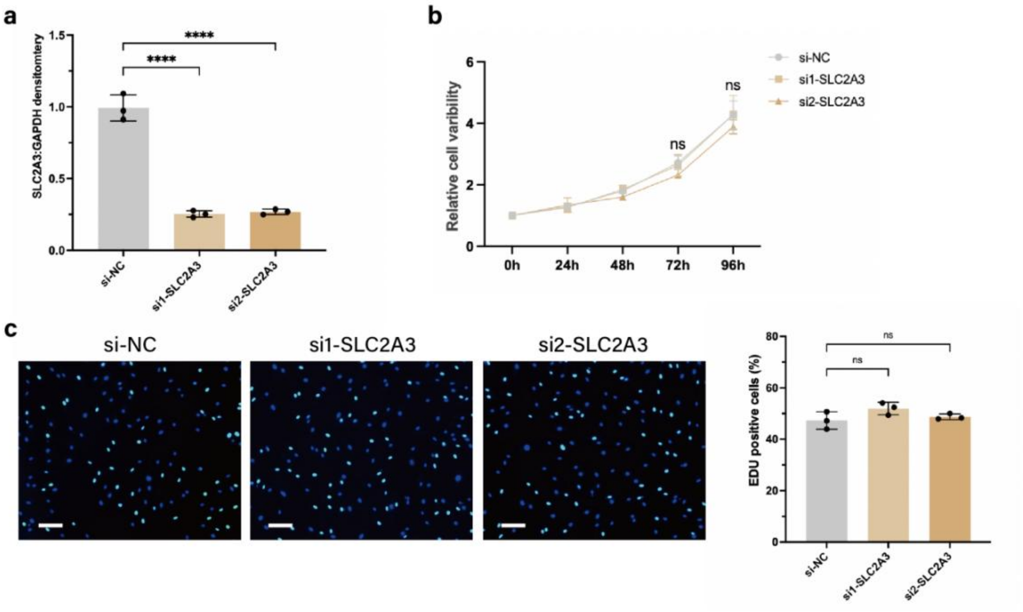

## Supplementary Figure S5

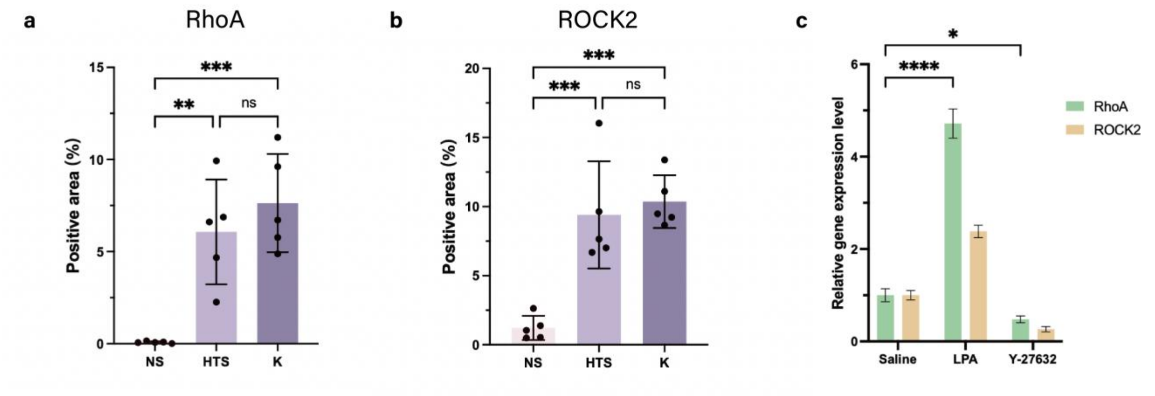


## Supplementary Figure S6

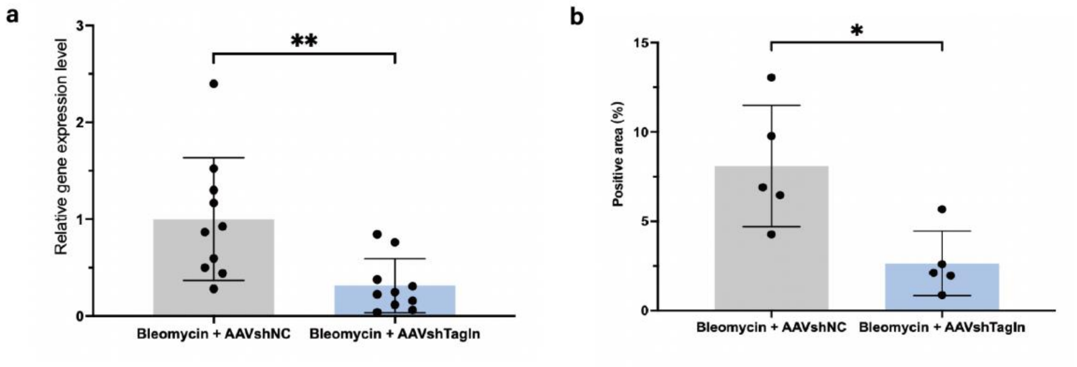

Supplement: Supplementary file 1 — Supplementary methods, figures and tables. [file ijbsv21p0658s1.pdf]
